# The Primary Responses of Murine Neonatal Lymph Node CD4^+^ Cells are Th2-skewed and are Sufficient for the Development of Th2-biased Memory

**DOI:** 10.1080/10446670310001598474

**Published:** 2003-03

**Authors:** Becky Adkins, Yurong Bu, Vladimir Vincek, Patricia Guevara

**Affiliations:** 1 Department of Microbiology and Immunology University of Miami Medical School Miami FL 33136 USA; 2 Department of Pathology University of Miami Medical School Miami FL 33136 USA

## Abstract

Exposure of neonatal mice to antigen often results in Th2-biased responses in later life. Examples of this Th2 tendency are (a) secondary antibody responses dominated by the Th2-associated IgG1 isotype and (b) Th2-mediated tolerance to alloantigens. We previously reported that neonates develop primary Th1 and Th2 function in the lymph nodes but exclusive Th2 primary splenic responses. Here, we have tested whether the Th2 bias of adults initially immunized as neonates is due to the early, primary Th2 polarization in the spleen. Surprisingly, removal of the spleen at birth had no affect on either IgG1-dominant secondary responses or the development of tolerance to alloantigens. Thus, neonatal lymph nodes are sufficient to generate Th2-biased function following neonatal antigen exposure. To understand how this could arise, we examined the primary Th1/Th2 responses of CD4^+^ lymph node cells. Unlike the balanced Th1/Th2 responses seen with total lymph node cells, the primary responses of isolated CD4^+^ cells were skewed to IL-4 producing function. These results suggest that the early development of Th2-dominant responses by lymph node CD4^+^ cells contributes substantially to the subsequent development of Th2-dominant memory in neonates.


*Clinical & Developmental Immunology*, March 2003, Vol. 10 (1), pp. 43–51

Taylor & Francis healthsciences

### The Primary Responses of Murine Neonatal Lymph Node CD4$^{+}$ Cells are Th2-skewed and are Sufficient for the Development of Th2-biased Memory

BECKY ADKINS[a,*], YURONG BU[a], VLADIMIR VINCEK[b] and PATRICIA GUEVARA[a]

[a]*Department of Microbiology and Immunology, University of Miami Medical School, Miami, FL 33136, USA;* [b]*Department of Pathology, University of Miami Medical School, Miami, FL 33136, USA*

Exposure of neonatal mice to antigen often results in Th2-biased responses in later life. Examples of this Th2 tendency are (a) secondary antibody responses dominated by the Th2-associated IgG1 isotype and (b) Th2-mediated tolerance to alloantigens. We previously reported that neonates develop primary Th1 and Th2 function in the lymph nodes but exclusive Th2 primary splenic responses. Here, we have tested whether the Th2 bias of adults initially immunized as neonates is due to the early, primary Th2 polarization in the spleen. Surprisingly, removal of the spleen at birth had no affect on either IgG1-dominant secondary responses or the development of tolerance to alloantigens. Thus, neonatal lymph nodes are sufficient to generate Th2-biased function following neonatal antigen exposure. To understand how this could arise, we examined the primary Th1/Th2 responses of CD4$^{+}$ lymph node cells. Unlike the balanced Th1/Th2 responses seen with total lymph node cells, the primary responses of isolated CD4$^{+}$ cells were skewed to IL-4 producing function. These results suggest that the early development of Th2-dominant responses by lymph node CD4$^{+}$ cells contributes substantially to the subsequent development of Th2-dominant memory in neonates.

*Keywords*: Ontogeny(neonatal); Th1/Th2; Memory; Tolerance; Spleen and lymph nodes

### INTRODUCTION

CD4$^{+}$ T helper (Th) cells can be functionally divided into Th1 and Th2 subsets (Mosmann and Coffman, 1989; Swain *et al.*, 1991; Abbas *et al.*, 1996). The Th1 subset produces -IFN and mediates delayed-type hypersensitivity and protection against intracellular pathogens. The Th2 subset produces IL-4 and IL-5 and is important in humoral responses. Immune responses heavily skewed toward Th1 or Th2 function can exacerbate infectious diseases, allergic reactions and autoimmunity. Thus, generating and maintaining the appropriate Th1/Th2 balance is critical for protective immunity. This is especially important for the very early stages of life, when exposure to many novel antigens occurs.

Over the last decade, it has become increasingly clear that murine neonates are immature in the development of Th function. In particular, murine neonates are prone to Th2 responses (reviewed in Adkins (2000)), Garcia *et al.* (2000) and Siegrist (2000). Notably, a single exposure to antigen during the neonatal period often leads to Th2 dominant memory responses much later in life, once the animals have grown to adulthood and are subsequently re-exposed to the same antigen. Well characterized examples of this Th2 bias are seen in the secondary responses to protein antigen immunization and in the maintenance of Th2-mediated tolerance to alloantigens.

The Th2 biased memory responses of animals originally immunized as neonates with protein antigen are clearly systemic. For example, memory effector cells from both the lymph nodes and spleens show preferential Th2 cytokine production when restimulated with the antigen *in vitro* (Barrios *et al.*, 1996; Adkins and Du, 1998). Moreover, neonates injected i.p. or i.v. with allogeneic cells subsequently reject, in a Th2-dependent manner, donor origin skin grafts transplanted to the tail in later adult life (Goldman *et al.*, 1989; Schurmans *et al.*, 1990; Chen *et al.*, 1995; Donckier *et al.*, 1995; Gao *et al.*, 1996). How these Th2 dominant responses originally arise is, at present, unclear. One possibility is that the Th1/Th2 pattern of memory cell generation is set early, during the primary response phase. For example, if neonates preferentially mounted Th2 primary responses initially, this might lead to relatively greater Th2 memory cell formation. In investigations of the primary responses of neonates, we have recently shown (Adkins *et al.*, 2000) that the responses that develop in the first week of life are

*Corresponding author. Tel.: +1-305-243-5560. Fax: +1-305-243-4623. E-mail: radkins@med.miami.edu

ISSN 1740-2522 print/ISSN 1740-2530 online © 2003 Taylor & Francis Ltd
DOI: 10.1080/10446670310001598474

dramatically different in the lymph nodes and spleen. While lymph node cells developed a mixed Th1/Th2 primary response to protein antigen immunization, neonatal spleens showed exclusive primary Th2 function.

In this report, we have addressed the hypothesis that the Th2-dominant primary responses in the neonatal spleen lead to the systemic Th2 dominant memory responses in later adult life. Spleens were surgically resected on day 1 of life and subsequent Th1/Th2 memory responses were monitored. As assessed by serum IgG responses and tolerance to skin allografts, quantitatively and qualitatively similar Th2-biased memory responses developed in the presence or absence of the spleen. Thus, the lymph node responses alone are sufficient to generate the secondary Th2 bias associated with neonatal exposure to antigen. Further analyses demonstrated that, while total lymph node cells developed balanced Th1/Th2 function in response to primary immunization, the primary responses of $CD4^+$ cells were skewed to the Th2 lineage. These results indicate that the preferential development of primary Th2 effector function in the neonatal lymph node $CD4^+$ population contributes substantially to the Th2 biased secondary responses of neonates.

### RESULTS

### The Neonatal Spleen is Not Required for the Development of Secondary Serum IgG Responses Dominated by the Th2-associated IgG1 Isotype

In an earlier study (Adkins *et al.*, 2001), we investigated the role of the neonatal spleen in the secondary cytokine recall responses of neonatal T cells. We found that animals splenectomized and immunized at birth developed Th2 dominant memory responses that were qualitatively and quantitatively similar to those produced by normal littermates. Those results suggested that the neonatal spleen is not required for the generation of Th2 dominant secondary responses. However, the possibility remained that the pattern of cytokines produced *in vitro* may not accurately reflect the levels of Th1/Th2 cytokines available *in vivo*. To address this issue, we chose to examine the relative levels of Th1- vs. Th2-associated IgG isotypes produced *in vivo* in response to T cell dependent antigen immunization. One day old or adult BALB/c mice were splenectomized and immunized with DNP-KLH in PBS. Normal, unmanipulated animals of each age group were immunized in parallel. Two weeks later, the animals were reimmunized with DNP-KLH in PBS. After an additional 2 weeks, sera were prepared and anti-DNP IgG2a (Th1-associated) and IgG1 (Th2-associated) levels were measured by specific ELISA (Fig. 1). As previously reported (Adkins and Du, 1998), the titers of secondary anti-DNP specific IgG1 antibodies were similar in normal neonates and adults while neonates produced markedly less specific IgG2a. This pattern was unaltered by splenectomy, either in neonates or adults.

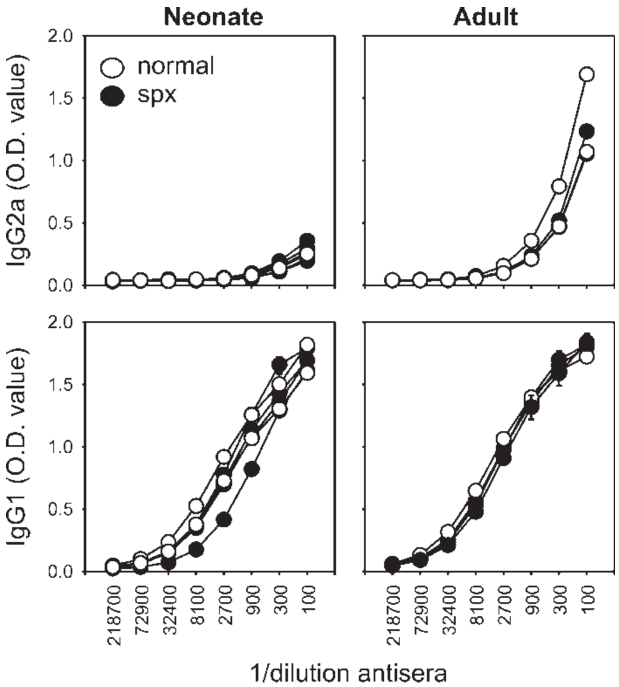

FIGURE 1 Removal of the spleen at birth does not diminish the dominance of the Th2-associated IgG1 isotype following secondary immunization. One day old or adult BALB/c mice were splenectomized and immunized with DNP-KLH in PBS. Normal, age-matched animals were also immunized in parallel. Two weeks later, all animals were reimmunized with DNP-KLH in PBS. Following an additional 2 weeks, sera were prepared and the levels of anti-DNP IgG2a and IgG1 were determined by specific ELISA. Each line represents an individual animal. Numbers of animals examined were as follows: normal neonates, 2; normal adults, 2; splenectomized (spx) neonates, 4; splenectomized adults, 2. All animals are shown; in some cases, it is not possible to distinguish individual normal animals (open circles) because their lines are superimposed with the closed circles. Each point is the average O.D. ± standard deviation of three replicate samples. Where error bars do not appear, the size of the error was smaller than the size of the symbol.

Thus, the neonatal lymph node response alone is sufficient to produce secondary responses dominated by the Th2-associated isotype IgG1.

### Neonatal Lymph Nodes are Sufficient to Generate and Maintain Th2-mediated Tolerance to Alloantigens as well as Tolerance to Peripheral Tissue Antigens

One of the unique hallmarks of the neonatal period is the capacity to become tolerant to allogeneic cells (Billingham *et al.*, 1953). It has been shown that IL-4 production *in vivo* is necessary for the induction and maintenance of neonatally induced tolerance to semiallogeneic F1 cells (Schurmans *et al.*, 1990; Donckier *et al.*, 1995; Gao *et al.*, 1996). Thus, we next tested whether the splenic Th2 dominant responses are required for the induction of tolerance to alloantigens. Four groups of 1-day-old BALB/c neonates were created. Group 1 was left completely unmanipulated (normals). The neonates in group 2 were splenectomized (spx). Group 3 animals were

TABLE I The spleen is not required for the generation of tolerance to alloantigens in neonates

| Group* | Skin graft | Number of rejected/number of transplanted | Mean day of rejection |
|---|---|---|---|
| Normal | CAF1 | 7/7 | 14.3 ± 3.1 |
| Spx | CAF1 | 8/8 | 18.9 ± 2.0 |
| Normal + CAF1 | CAF1 | 1/4 | Not applicable |
| Spx + CAF1 | CAF1 | 0/7 | Not applicable |
| Normal | C57BL/6 | 7/7 | 14.0 ± 3.3 |
| Spx | C57BL/6 | 8/8 | 18.3 ± 2.1 |
| Normal + CAF1 | C57BL/6 | 4/4 | 17.6 ± 3.2 |
| Spx + CAF1 | C57BL/6 | 7/7 | 16.9 ± 2.9 |

* One group of 1 day old BALB/c mice was splenectomized (spx); another group was not splenectomized. Each of these two groups was divided into two groups; one set received $1 \times 10^7$ CAF1 spleen plus lymph node cells i.v. the other set received no cells. Four weeks later, all animals were transplanted with skin grafts from CAF1 or C57BL/6 mice. The survival of the skin grafts was subsequently monitored for 42 days.

injected i.v. with $1 \times 10^7$ spleen and lymph node cells from adult CAF1 mice (normal + CAF1). Group 4 animals were splenectomized and injected with adult CAF1 cells (spx + CAF1). Four weeks later, all animals received skin grafts from donor-type CAF1 mice or from third party, unrelated C57BL/6 mice. The survival of the grafts was then recorded daily, out to 42 days post grafting (Table I). All animals, regardless of their treatment during neonatal life, rejected the third party C57BL/6 skin grafts within similar time spans. Animals splenectomized as neonates rejected CAF1 grafts as readily as their normal littermates. Surprisingly, the absence of the spleen had no effect on the capacity of neonates to become tolerant to CAF1 cells. While 1 of 4 mice in the normal group that received CAF1 cells at birth actually rejected the CAF1 graft, all of the seven splenectomized neonates that received CAF1 cells at birth retained their grafts for 42 days, with no signs of rejection. Thus, the responses generated in the neonatal lymph nodes alone are sufficient to establish and maintain neonatal tolerance to alloantigens.

In normal neonates, Th2 responses have been shown to dominate in this transplantation model (Goldman *et al.*, 1989; Schurmans *et al.*, 1990; Chen *et al.*, 1995; Donckier *et al.*, 1995; Gao *et al.*, 1996). To determine whether exclusive Th2 responses also developed in the absence of the spleen, the Th1/Th2 recall responses of lymph node cells from the same animals were monitored *in vitro* 49 days after the initial skin grafting. As expected from the normal rejection of the C57BL/6 skin grafts, all groups mounted a robust and exclusive Th1 response to C57BL/6 splenocytes (Fig. 2). Both the normal and the splenectomy groups also produced high levels of -IFN but no IL-4 in response to restimulation with CAF1 cells. These results are expected since these animals were not exposed to CAF1 or C57BL/6 cells as neonates but were primed as adults to CAF1 and C57BL/6 antigens via the skin grafts. For all of the normal animals that received CAF1 cells at birth, IFN production in response to CAF1 cells was low to undetectable. Two of the four animals in this group produced high levels of IL-4 while IL-4 was undetectable in the other two. Similarly, in the group that was splenectomized and exposed to CAF1 cells at birth, 3 of the 7 animals produced high levels of IL-4 in response to CAF1 cells. However, unlike in the corresponding normal group, two of the animals in this group also made high levels of IFN. These results suggest that secondary responses arising in asplenic neonates may not always be exclusively Th2 but may instead be mixed Th1/Th2, similar to the primary responses seen in neonatal lymph nodes. Nonetheless, because these animals all became tolerant to CAF1 cells, as evidenced by acceptance of CAF1 skin grafts, the Th2 response (or no response) must be dominant *in vivo*.

While tolerance to injected alloantigens does not appear to require the spleen, it seemed possible that other forms of tolerance that must be established in early life may require the neonatal spleen. Notably, in neonates, a large number of newly generated T cells are encountering peripheral tissue antigens for the first time. Tolerance to these antigens must be established in the periphery to ensure that autoimmunity does not develop. Since T cell mediated autoimmunity is often a Th1-associated phenomenon (Liblau *et al.*, 1995), it could be postulated that the Th2 dominant responses of the neonatal spleen act to suppress Th1 activity and hence, help to avoid autoimmune reactions. A clear prediction of this hypothesis is that removal of the early splenic Th2 responses would lead to autoimmune reactions to cells or organs in the periphery. This idea was tested by assessing the potential for the development of autoreactivity in splenectomized neonates. Control and splenectomized neonates were allowed to grow to 6 months of age. At that time, major organs that are common targets in autoimmune disease were examined histologically for gross signs of tissue pathology. The pancreas, stomachs, thyroids, kidneys, adrenals, thymus, colons and testes and ovaries of the splenectomized mice were indistinguishable from those of the control normal mice (data not shown). Moreover, the sera from the splenectomized mice did not contain elevated levels of autoantibodies commonly found in autoimmune disease (Fig. 3).

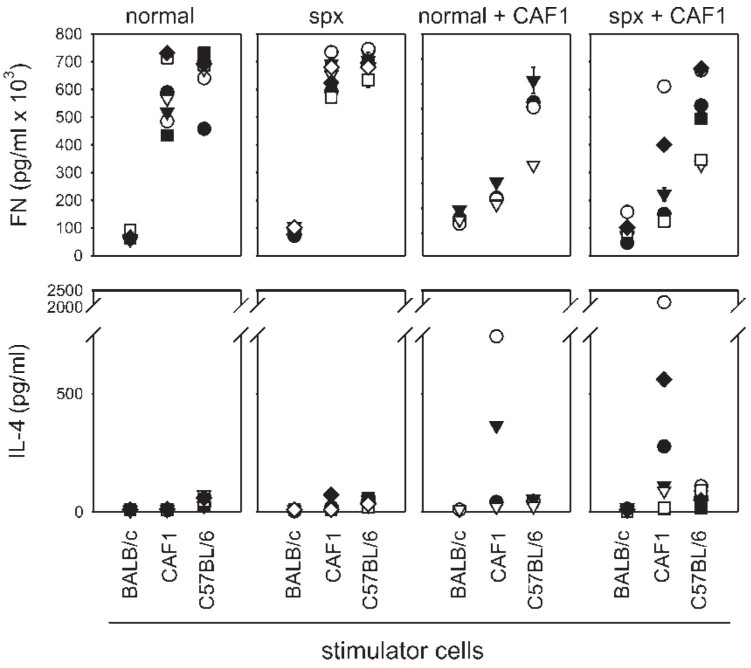

FIGURE 2 Th1/Th2 cytokine production by normal and splenectomized animals injected at birth with semiallogeneic F1 cells. Four groups of 1 day old BALB/c neonates were analyzed. Group 1 was unmanipulated (normals). Neonates in group 2 were splenectomized (spx). Group 3 neonates were injected i.v. with $1 \times 10^7$ spleen and lymph node cells from adult CAF1 mice (normal + CAF1). Group 4 neonates were splenectomized and injected with adult CAF1 cells (spx + CAF1). Four weeks later, all animals received skin grafts from donor-type CAF1 mice or from third party, unrelated C57BL/6 mice. Forty-nine days later, lymph node cells were restimulated with mitomycin treated splenocytes from syngeneic BALB/c mice or from CAF1 or C57BL/6 mice. Supernatants were tested for IFN (48 h supernatants) or IL-4 (72 h supernatants) content by specific ELISA. Each symbol represents an individual animal. The numbers of animals in each group were as follows: normal, 7; spx, 8; normal + CAF1, 4; spx + CAF1, 7. Each point is the average ± standard deviation from triplicate samples. Where error bars do not appear, the size of the error was smaller than the size of the symbol.

Thus, like tolerance to alloantigens, the lymph nodes of neonates are sufficient to establish peripheral tolerance to tissue antigens in early life.

### Balanced Frequencies of Primary IL-4: IFN Secreting Cells among Total Neonatal Lymph Node Cells but Th2 Skewing among the CD4$^+$ Population

The previous experiments clearly demonstrated that neonates develop Th2 skewed secondary responses, even in the absence of the Th2 dominant primary responses of the spleen. For Th2 dominant memory responses to become established in neonates, the evidence suggests that Th2 responses must dominate early, at least in the allogeneic tolerance model we have used (Schurmans *et al.*, 1990; Donckier *et al.*, 1995; Gao *et al.*, 1996). Together with our splenectomy experiments, this would suggest that Th2 dominance must be established during primary responses in the lymph nodes. However, in earlier studies (Adkins and Du, 1998), we had found that primary lymph node responses were not Th2 skewed, showing instead the same relative production of Th1/Th2 cytokines as in adult lymph nodes. In these earlier experiments, we used ELISA assays, which measure the total amount of cytokine produced in bulk cultures *in vitro*. We next considered the possibility that Th2 dominance in the neonatal lymph nodes may be achieved by the development of greater numbers of primary Th2 compared to Th1 cells. To test this possibility, we used ELISPOT assays to measure the frequencies of primary - IFN- or IL-4 secreting cells in neonatal vs. adult lymph nodes. One day old or adult BALB/c mice were immunized with KLH in PBS and the frequencies of IFN- or IL-4-secreting cells were determined 1 week later by ELISPOT (Table II). Greater frequencies (2–8 fold higher; $p < 0.001$ within each experiment) of both IFN- and IL-4-producing cells developed in neonatal compared to adult lymph nodes. Nonetheless, consistent with our earlier ELISA data (Adkins and Du, 1998), the ratios of IL-4: IFN-secreting cells among neonatal cells were not significantly different from those of adult cells (Table II).

The secondary responses we have analyzed, serum IgG memory responses and alloantigen tolerance, are both mediated by CD4$^+$ cells. Therefore, we wished to examine further the primary responses in the lymph nodes by investigating the functions of isolated CD4$^+$ cells. One day old or adult BALB/c mice were immunized with KLH in PBS. One week later, lymph node CD4$^+$ cells were prepared, re-stimulated with KLH in the presence of adult splenic APC, and assessed for the frequencies of IFN- or IL-4-secreting cells by ELISPOT (Fig. 4). Similar frequencies of IFN-secreting cells were seen among CD4$^+$ cells from neonatal and adult lymph nodes. In contrast, significantly greater ($p < 0.001$) frequencies

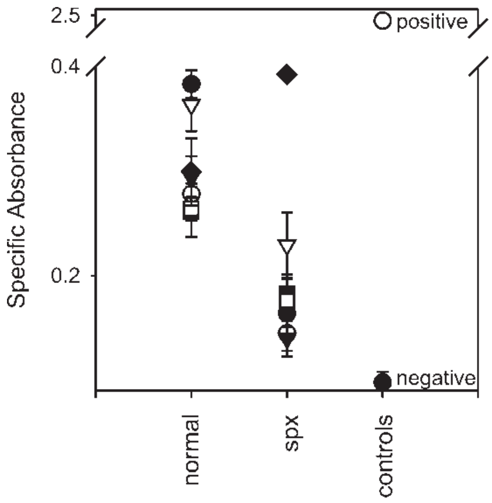

FIGURE 3 Removal of the spleen at birth does not result in increased incidence of autoantibody production. One day old BALB/c mice were left unmanipulated (normal) or splenectomized (spx). Six months later, sera were prepared and tested for anti-nuclear antibodies using commercially purchased ELISA kits, according to the manufacturer's directions. The specific absorbance was calculated as the absorbance values obtained with samples placed in antigen-coated wells minus the absorbance values obtained with samples placed in non-coated wells. Positive and negative controls were provided by the manufacturer. The numbers of animals were as follows: normal, 7; splenectomized, 7. Each point is the average ± standard deviation from triplicate samples of individual animals.

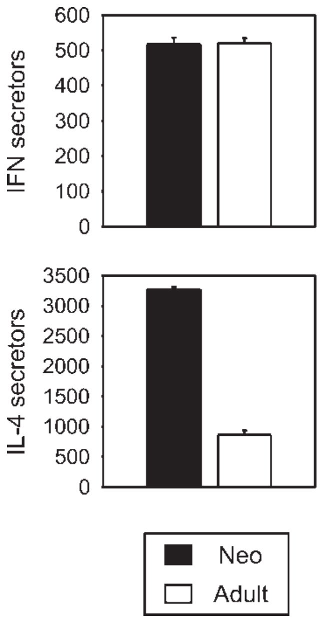

FIGURE 4 Th2-skewed CD4$^+$ primary responses develop in neonatal lymph nodes in response to protein antigen immunization. One day old or adult BALB/c mice were immunized with KLH in PBS. One week later, CD4$^+$cells were prepared from lymph nodes and restimulated with KLH in the presence of adult splenic APC. After 48 h, the cells were processed by ELISPOT methods to determine the frequencies of IFN- or IL-4-secreting cells. One experiment typical of two separate experiments for each responding cell type is shown. The frequencies of cytokine-secreting cells (y axis) are per $10^6$ CD4$^+$ cells.

of IL-4-secreting cells were seen among CD4$^+$ cells from neonates, compared to adult CD4$^+$ cells. Thus, there is clear Th2-skewing among neonatal CD4$^+$ cells in the primary response to protein antigen immunization.

To ensure that Th2-skewed primary responses among neonatal CD4$^+$ cells were not limited to protein antigens, primary responses to alloantigen immunization were similarly examined. One day old neonatal or adult BALB/c mice were injected with $1 \times 10^7$ spleen and lymph node cells from adult CAF1 mice. Parallel, aged matched mice that did not receive any cells served as control, non-primed mice. One week later, CD4$^+$ cells were prepared from lymph nodes and restimulated with mitomycin C treated CAF1 spleen cells. The IFN and IL-4 contents of the supernatants were analyzed by ELISA (Fig. 5). IFN was readily detected in the supernatants from both the neonatal and adult cultures; neonatal CD4$^+$ lymph node cells made approximately 2-fold less IFN than did adult cells. High levels of IL-4 were also secreted by neonatal CD4$^+$ cells. In contrast, IL-4 was undetectable in the adult CD4$^+$ cell cultures. Thus, similar to what was observed with protein antigen immunization, neonatal CD4$^+$ cells developed a mixed Th1/Th2 primary response to alloantigen immunization and the neonatal response was clearly Th2-skewed, compared to the adult response.

To test whether neonatal APC function was important in regulating Th2-skewed responses, the *in vitro* responses of isolated neonatal CD4$^+$ cells were examined. CD4$^+$ cells were prepared from unprimed 7-day-old or adult BALB/c mice and stimulated in culture with mitomycin C treated splenocytes from either fully allogeneic (C57BL/6) or semi-allogeneic (CAF1) adult mice. Supernatents were harvested at 24, 48 and 72 h of culture and the secreted cytokines were measured by ELISA (Fig. 6). In both

TABLE II Higher frequencies of both IFN- or IL-4-secreting cells develop in neonatal, compared to adult lymph nodes, in response to primary immunization

| | Per $10^6$ total lymph node cells* | | |
|---|---|---|---|
| | IFN secretors | IL-4 secretors | Ratio of IL-4: IFN secretors |
| Exp 1 | | | |
| Neo | 773.3 ± 111.5 | 3,462.7 ± 360.7 | 4.48 |
| Adult | 125.8 ± 10.1 | 450.0 ± 40.1 | 3.58 |
| Exp 2 | | | |
| Neo | 1,128.8 ± 133.0 | 4,618.4 ± 563.7 | 4.09 |
| Adult | 660.0 ± 14.1 | 1,677.5 ± 242.7 | 2.54 |
| Exp 3 | | | |
| Neo | 1,983.3 ± 76.5 | 6,416.7 ± 58.9 | 3.24 |
| Adult | 790.0 ± 50.0 | 2,018.8 ± 106.1 | 2.56 |
| Exp 4 | | | |
| Neo | 1,017.1 ± 0.6 | 2,446.1 ± 108.3 | 2.40 |
| Adult | 236.0 ± 10.6 | 1,187.1 ± 58.6 | 5.03 |

* One day old or adult BALB/c mice were immunized with KLH in PBS. One week later, total lymph node cells were prepared and assayed by ELISPOT analyses for the frequencies of KLH specific, IFN- or IL-4-secreting cells. Frequencies are averages ± standard deviations from four replicate samples.

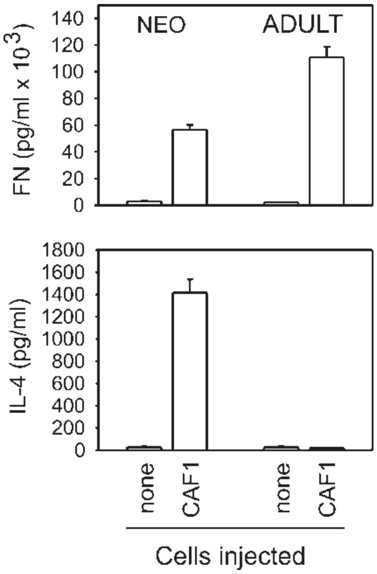

FIGURE 5 Neonatal lymph node CD4$^+$ cells develop Th2-skewed primary function *in vivo* in response to alloantigen exposure. One day old or adult BALB/c mice were injected i.v. with $1 \times 10^7$ spleen and lymph node cells from adult CAF1 mice. Age-matched control mice did not receive any cells. One week later, CD4$^+$ lymph node cells were prepared and stimulated with mitomycin C treated CAF1 splenocytes. Supernatants were harvested 48 h later and IFN and IL-4 content were measured by specific ELISA. One experiment representative of two independent experiments is shown.

settings, neonatal CD4$^+$ cells demonstrated Th2-skewed responses, with patterns of cytokine production similar to those that developed *in vivo*. Thus, the development of Th2-biased primary responses does not appear to require neonatal APC. Together, these results indicate that lymph node CD4$^+$ cells in neonates develop Th2 biased primary responses; these biased primary responses may contribute substantially to the Th2-skewed secondary responses observed both in normal and splenectomized neonates.

### DISCUSSION

We have addressed the hypothesis that the exclusive Th2 primary responses that develop in the neonatal spleen are responsible for the Th2-biased secondary responses often seen in neonates. Two experimental systems in which Th2-skewing occurs in response to neonatal antigen exposure were used: (a) protein immunization leading to secondary antibody responses dominated by the Th2 isotype IgG1 and (b) the injection of allogeneic cells resulting in Th2-mediated tolerance to alloantigens. These responses were compared in normal neonates and in neonates in which the spleen was removed at birth. Surprisingly, the absence of the spleen had no effect on these responses; splenectomized neonates were equivalent to their normal littermates in the development of secondary IgG1-dominant serum responses and tolerance to alloantigens. In addition, the neonatal period is characterized by the natural establishment of tolerance to self-antigens found in peripheral organs. Histological and serum autoantibody screening showed that removal of the spleen at birth also did not increase the incidence of autoimmunity. Thus, responses occurring in the neonatal lymph nodes alone are sufficient to establish Th2 skewed secondary responses to protein antigen immunization as well as tolerance to allo- and self-antigens. To understand how Th2 skewed secondary responses become

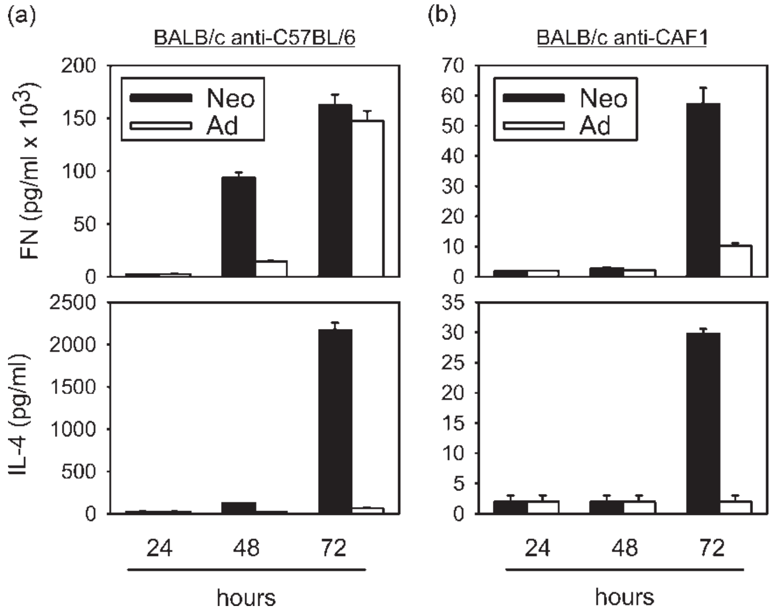

FIGURE 6 Neonatal lymph node CD4$^+$ cells develop Th2-skewed primary function *in vitro*, in the absence of neonatal APC. CD4$^+$ cells were prepared from lymph nodes of 7 day old or adult unprimed BALB/c mice. The cells were cocultured with mitomycin C treated splenocytes from C57BL/6 (a) or CAF1 (b) adult mice. Supernatants were collected at 24, 48 and 72 h cytokine content was analyzed by specific ELISA.

established, we examined the frequencies of IFN- or IL-4-secreting cells developing during primary responses in neonatal lymph nodes. ELISPOT analyses revealed that significantly greater frequencies of both IFN- and IL-4-secreting cells developed among total neonatal, compared to adult, lymph node cells. Greater frequencies of IL-4-producing cells also developed among isolated $CD4^+$ neonatal cells, compared to adult $CD4^+$ cells. In contrast, $CD4^+$ neonatal cells developed frequencies of IFN-secreting cells similar to those found among the $CD4^+$ adult population. As a result, while total lymph node cells in neonates and adults showed similar ratios of IL-4: IFN secretors, this ratio for neonatal $CD4^+$ population was skewed to IL-4 production.

One striking observation in these experiments is that the absolute amounts of secondary serum antibodies appear to be similar in normal and splenectomized neonates. Since the lymph nodes are the only site of B cell memory development in splenectomized neonates, the lymph nodes may then also be the major site of B cell memory formation in intact neonates. B cell memory development is promoted by primary $CD4^+$ cells that home to germinal centers (reviewed in MacLennan (1994), Przylepa *et al.* (1998) and Tarlinton (1998)). Therefore, the production of Th2-skewed B cell memory responses, even in the absence of the spleen, is consistent with the development of primary lymph node $CD4^+$ responses that are Th2-biased. We previously reported (Adkins and Du, 1998) that, although secondary B cell responses are Th2-biased, neonates and adults generate similar ratios of the IgG2a (Th1-associated) and IgG1 (Th2-associated) isotypes in primary serum responses. If primary $CD4^+$ responses are Th2-skewed, how do primary serum IgG2a/IgG1 responses that are adult-like arise? One possibility is that, unlike in germinal centers, non-$CD4^+$ cells may provide a more balanced (or even Th1-skewed) source of cytokines for usage by primary B cell antibody forming cells in the T cell zones. Thus, we are examining the Tc1/Tc2 responses of neonatal $CD8^+$ cells to determine whether $CD8^+$ cells might provide more balanced levels of IFN/IL-4 during primary responses in neonatal lymph nodes.

In earlier studies (Adkins *et al.*, 2000), we showed that primary effector responses arising in the neonatal spleen were exclusively Th2-like. This report establishes that the primary responses among neonatal lymph node $CD4^+$ cells, while not exclusively Th2, are biased to the Th2 lineage. How could this arise? In adults, it is generally believed that the major factors controlling Th1 vs. Th2 development are the environmental signals (cytokines, costimulation) present at the time of initial priming (Swain *et al.*, 1991; Seder and Paul, 1994; Abbas *et al.*, 1996; O'Garra, 1998; Moser and Murphy, 2000). Thus, signals coming from the APC compartment or other cell types may result in Th2 skewed development in neonates. Some recent evidence is consistent with this possibility. For example, reduced expression of class II MHC and costimulatory molecules have been reported for murine neonatal dendritic cells, B cells and monocytes (Goriely *et al.*, 2001; Muthukkumar *et al.*, 2002). Diminished production of the Th1 promoting cytokine IL-12 by neonatal monocytes has also been described (Goriely *et al.*, 2001). In contrast to those reports, however, phenotypically mature dendritic cells capable of promoting vigorous CTL responses (and presumably strong Th1 responses) have recently been isolated from the neonatal spleen, albeit in reduced numbers (Dadaglio *et al.*, 2002). Thus, at this point, the contribution of APC function to neonatal Th2 skewing is unclear. Another possibility is that intrinsic properties of the T cells themselves are a factor in neonatal Th2 skewing. Our data showing that Th2-skewing develops *in vitro*, in the presence of adult APC (Fig. 6), is consistent with this idea. Moreover, we recently reported (Adkins *et al.*, 2002) that intrinsic properties of neonatal T cells contribute to Th2-skewing *in vivo*; in adoptive adult hosts, neonatal lymph node $CD4^+$ cells were highly deficient in the development of Th1 function. How might these functional differences between neonatal and adult $CD4^+$ cells arise? There is at least one clear way in which naive neonatal and adult T cell populations differ. In the adult, only a very small percentage of peripheral T cells are recently emigrated from the thymus. In contrast, the vast majority of peripheral T cells in neonates are new thymic emigrants. In fact, the primary effector functions of lymph node $CD4^+$ cells in neonates look remarkably similar to those of recent thymic migrants in adults. In particular, mixed Th1/Th2 responses are elicited but, compared to resident peripheral adult T cells, the responses of recent migrants are Th2-skewed (Bendelac *et al.*, 1992). We are in the process of directly testing the idea that neonatal T cell Th function results from their recent migration from the thymus.

The experiments here as well as those reported earlier (Adkins *et al.*, 2001) clearly show that the neonatal spleen is not required for many of the Th2-mediated functions associated with neonatal antigen exposure. Why neonatal primary splenic responses would be so Th2-biased is still a mystery. One possibility is that the neonatal splenic Th2 responses are only a part of the machinery necessary for generating, e.g. tolerance to allo- or self-antigens. In the absence of the spleen, other mechanisms (e.g. regulatory cells) may compensate to generate tolerance. If this is so, it may not be possible to see a major effect of eliminating the splenic responses. It may, however, be possible to see an augmentation or acceleration of one of these processes. For example, in a case where animals develop juvenile onset, Th1-mediated autoimmune disease, as in NOD mice (Yoshida and Kikutani, 2002), removal of the spleen may exacerbate the disease or accelerate disease onset. We are currently considering examining the effect of neonatal splenectomy in autoimmune disease models, such as the NOD mice.

Studies from the laboratory of M. Goldman (Donckier *et al.*, 1995) showed that IL-4 RNA is upregulated in neonatal lymph nodes as early as 2 days after the injection of allogeneic cells. Our results, examining the responses of lymph node cells one week after the injection of allogeneic cells, confirm this result and extend it in two ways. First, we have shown that high levels of IL-4 protein are made by neonatal, but not adult, $CD4^+$ lymph node cells. Second, we demonstrate that, in the primary response phase, significant alloantigen-specific Th1 activity also develops. However, this primary Th1 function is apparently not converted to or maintained as memory very efficiently because, in normal neonates, little to no IFN is produced by alloantigen-specific memory cells. How this occurs is unclear but we know from *in vitro* experiments that neonatal T cells are more susceptible to apoptosis than adult T cells (Adkins *et al.*, 1996). Thus, it is tempting to speculate that the Th1 cells arising in neonates are more susceptible than the Th2 cells to apoptotis induction.

### MATERIALS AND METHODS

### Mice

BALB/c mice, originally obtained from Charles River Laboratories (Wilmington, MA), were bred and housed under barrier conditions in the Division of Veterinary Resources at the University of Miami Medical School. Periodic screening showed the colony to be free of commonly occuring infectious agents. Females from timed matings were monitored closely from days 19–21 of gestation and the date of delivery recorded. Birth day was called day 0. Neonatal animals were defined as 1 day old.

C57BL/6 and (BALB/c × A/J)F1 mice, referred to as CAF1 mice, were purchased from Jackson Laboratories (Bar Harbor, ME). Adult (>6 weeks old) mice of these strains were used for donor cells and skin.

### Protein Antigen Immunization

One day old neonatal or adult (6–10 weeks old) BALB/c mice were immunized with, respectively, 10 or 100 μg KLH (CalBiochem, San Diego, CA) or DNP-KLH (CalBiochem) in PBS. Each mouse was injected in three sites—intraperitoneally (i.p.) and subcutaneously (s.c.) between the shoulder blades and at the base of the tail. Adults received 100 μl and neonates 10 μl per site. For the experiment shown in Fig. 1, the animals were reimmunized with DNP-KLH in PBS 2 weeks after the initial immunization. Adults again received 100 μg; the 2 week old mice originally immunized as neonates were weighed and re-immunized with 5 μg/g DNP-KLH.

### Injection of CAF1 Cells into Neonates

Adult CAF1 mice were used to prepare donor cells. Red cells were removed from spleen cell suspensions by hypotonic lysis and mesenteric, inguinal, axillary, brachial and cervical lymph nodes were pooled to prepare lymph node cell suspensions (Adkins and Du, 1998; Adkins *et al.*, 2000). Preparations of spleen and lymph node cells were mixed and washed twice with HBSS prior to injection. One day old BALB/c neonates or adult mice were injected with $1 \times 10^7$ pooled spleen and lymph node cells; neonates received a total volume of 50 μl in the facial vein, adults were injected with a total volume of 250 μl in the tail vein.

### Splenectomy

Neonates and adults were splenectomized as described previously (Adkins *et al.*, 2001). Briefly, anesthetized animals were placed on their stomachs and a small incision was made on the right back side, just below the rib cage. Spleens were pinched off with forceps and the wounds were closed with silk braided sutures (Ethicon, attached 5-0 needle) for neonates or with surgical staples for adults.

### Skin Grafting

Using a number 11 scalpel, sections of epidermis approximately 4 mm long by 2 mm wide were removed from the tails of anesthetized donors and host animals. Donor skin was placed on a reciprocal, prepared site on the host tails, in reverse orientation so that hair growth was in the opposite direction from that of the host. Slight pressure was applied with sterile gauze to allow for initial adherence. The tails were left in the open air for 10–15 min. To protect the graft site, plastic tubes with air holes and with inner diameters approximately twice that of the diameter of the tails were placed over the tails. The tubes were held in place with masking tape made into butterfly shapes; the "butterflies" were taped to the tails just beneath the tubes. Mice with skin grafts were housed in cages with corncob or paper towel bedding. The tubes were removed on day 2–3 after grafting.

Beginning on day 4, the grafts were judged for signs of rejection. Complete rejection was defined as shriveling of the graft leading to the drying and flaking away of the graft. Graft acceptance was defined as a completely healed graft that was differentiated from the rest of the tail skin by the reverse growth of the graft hair.

### Serum ELISA for IgG1 and IgG2a Isotype

Serum samples from individual animals were analyzed separately using IgG1 or IgG2a specific ELISA assays, as previously described (Adkins and Du, 1998).

### ELISA for Anti-nuclear Antibodies

Serum samples from different animals were analyzed individually using an anti-nuclear antibody kit (Alpha Diagnostic, San Antonio, TX), precisely per the manufacturer's instructions.

### Preparation of Cells for Tissue Culture

Total spleen cell suspensions were prepared and RBC were removed by incubation in hypotonic lysis buffer (0.15 M $NH_4Cl$, 0.001 M $KHCO_3$ and 0.1 mM EDTA). Mesenteric, inguinal, axillary, brachial and cervical lymph nodes were pooled and used for total lymph node cell suspensions.

Enriched $CD4^+$ (95–98% $CD4^+$) cells were positively selected ($MS^+$ columns) using the Miltenyi Biotec (Bergisch-Gladbach, Germany) MACS system, according to the manufacturer's directions.

For examining allogeneic responses (Figs. 2 and 5), stimulator cells were spleen cells from C57BL/6, BALB/c or CAF1 mice treated with 50 μg/ml mitomycin C. For stimulation of $CD4^+$ cells with KLH (Fig. 4), splenic APC were prepared by treating total BALB/c spleen cells with anti-Thy-1 (mAb 42-21) plus complement, followed by treatment with 50 μg/ml mitomycin C.

### Cytokine ELISA and ELISPOT Assays

The culture conditions and the methods for assaying cytokine production by or cytokine secreting cells among total lymph node and spleen cells in response to restimulation with KLH have been previously described in detail (Adkins and Du, 1998; Adkins *et al.*, 2000; Adkins *et al.*, 2001).

For the experiments analyzing cytokine production by alloantigen specific memory cells (Fig. 2), total lymph node cells from individual animals were cultured separately. $2.5 \times 10^5$ lymph node responders and $5.0 \times 10^5$ splenic stimulator cells were cultured in 200 μl of medium per well in 96 well plates. For the experiments analyzing primary responses of fractionated cells (Figs. 4–6), $CD4^+$ cells were cultured at $2.0 \times 10^5$ cells per well. $5.0 \times 10^5$ CAF1 or C57BL/6 splenic stimulator cells were used for the alloantigen stimulation (Figs. 5 and 6) or $5.0 \times 10^5$ BALB/c splenic APC were used for restimulation with 50 μg/ml KLH (Fig. 4).

### *Acknowledgements*

The authors would like to thank Dr Christa E. Müller-Sieburg (Sidney Kimmel Cancer Center, San Diego, CA) for stimulating discussions and critical evaluation of the manuscript. The authors would also like to thank Dr Carolyn Cray, Department of Pathology, University of Miami Medical School, for her guidance on the preparation and evaluation of skin grafts.

### *References*

Abbas, A.K., Murphy, K.M. and Sher, A. (1996) "Functional diversity of helper T lymphocytes", *Nature* **383**, 787–793.

Adkins, B. (2000) "Development of neonatal Th1/Th2 function", *Int. Rev. Immunol.* **19**, 157–171.

Adkins, B. and Du, R.-Q. (1998) "Newborn mice develop balanced Th1/Th2 primary effector responses *in vivo* but are biased to Th2 secondary responses", *J. Immunol.* **160**, 4217–4224.

Adkins, B., Chun, K., Hamilton, K. and Nassiri, M. (1996) "Naïve murine neonatal T cells undergo apoptosis in response to primary stimulation", *J. Immunol.* **157**, 1343–1349.

Adkins, B., Bu, Y., Cepero, E. and Perez, R. (2000) "Exclusive Th2 primary effector function in spleens but mixed Th1/Th2 function in lymph nodes of murine neonates", *J. Immunol.* **164**, 2347–2353.

Adkins, B., Bu, Y. and Guevara, P. (2001) "The generation of Th memory in neonates versus adults: prolonged primary Th2 effector function and impaired development of Th1 memory effector function in murine neonates", *J. Immunol.* **166**, 918–925.

Adkins, B., Bu, Y. and Guevara, P. (2002) "Murine neonatal $CD4^+$ lymph node cells are highly deficient in the development of antigen-specific Th1 function in adoptive adult hosts", *J. Immunol.* **169**, 4998–5004.

Barrios, C., Brawand, P., Berney, M., Brandt, C., Lambert, P.H. and Siegrist, C.A. (1996) "Neonatal and early life immune responses to various forms of vaccine antigens qualitatively differ from adult responses: predominance of a Th2-biased pattern which persists after adult boosting", *Eur. J. Immunol.* **26**, 1489–1496.

Bendelac, A., Matzinger, P., Seder, R.A., Paul, W.E. and Schwartz, R.H. (1992) "Activation events during thymic selection", *J. Exp. Med.* **175**, 731–742.

Billingham, R.E., Brent, L. and Medawar, P.B. (1953) "Actively acquired tolerance of foreign cells", *Nature* **172**, 603–605.

Chen, N., Gao, Q. and Field, E.H. (1995) "Expansion of memory Th2 cells over Th1 cells in neonatal primed mice", *Transplantation* **60**, 1187–1193.

Dadaglio, G., Sun, C.M., Lo-Man, R., Siegrist, C.A. and le Clerc, C. (2002) "Efficient *in vivo* priming of specific cytotoxic T cell responses by neonatal dendritic cells", *J. Immunol.* **168**, 2219–2224.

Donckier, V., Wissing, M., Bruyns, C., Abramowicz, D., Lybin, M., van der Haeghen, M.-L. and Goldman, M. (1995) "Critical role of interleukin 4 in the induction of neonatal transplantation tolerance", *Transplantation* **59**, 1571–1576.

Gao, Q., Chen, N., Rouse, T.M. and Field, E.H. (1996) "The role of interleukin-4 in the induction phase of allogeneic neonatal tolerance", *Transplantation* **62**, 1847–1854.

Garcia, A.M., Fadel, S.A., Cao, S. and Sarzotti, M. (2000) "T cell immunity in neonates", *Immunol. Res.* **22**, 177–190.

Goldman, M., van der Vorst, P., Lambert, P., Doutrelepont, J.-M., Bruyns, C. and Abramowicz, D. (1989) "Persistence of anti-donor helper T cells secreting interleukin 4 after neonatal induction of transplantation tolerance", *Transplant. Proc.* **21**, 238–239.

Goriely, S., Vincart, B., Stordeur, P., Vekemans, J., Willems, F., Goldman, M. and de Wit, D. (2001) "Deficient IL-12 (p35) gene expression by dendritic cells derived from neonatal monocytes", *J. Immunol.* **166**, 2141–2146.

Liblau, R.S., Singer, S.M. and McDevitt, H.O. (1995) "Th1 and Th2 $CD4^+$ T cells in the pathogenesis of organ-specific autoimmune diseases", *Immunol. Today* **16**, 34–38.

MacLennan, I.C. (1994) "Germinal centers", *Annu. Rev. Immunol.* **12**, 117–139.

Moser, M. and Murphy, K.M. (2000) "Dendritic cell regulation of TH1–TH2 development", *Nat. Immunol.* **1**, 199–205.

Mosmann, T.R. and Coffman, R.L. (1989) "TH1 and TH2 cells: different patterns of lymphokine secretion lead to different functional properties", *Annu. Rev. Immunol.* **7**, 145.

Muthukkumar, S., Goldstein, J. and Stein, K.E. (2002) "The ability of B cells and dendritic cells to present antigen increases during ontogeny", *J. Immunol.* **165**, 4803–4813.

O'Garra, A. (1998) "Cytokines induce the development of functionally heterogeneous T helper cell subsets", *Immunity* **8**, 275–283.

Przylepa, J., Himes, C. and Kelsoe, G. (1998) "Lymphocyte development and selection in germinal centers", *Curr. Top. Microbiol. Immunol.* **229**, 85–104.

Schurmans, S., Heusser, C.H., Qin, H.-Y., Merino, J., Brighouse, G. and Lambert, P.-L. (1990) "*In vivo* effects of anti-IL-4 monoclonal antibody on neonatal induction of tolerance and on an associated autoimmune syndrome", *J. Immunol.* **145**, 2465–2473.

Seder, R.A. and Paul, W.E. (1994) "Acquisition of lymphokine-producing phenotype by $CD4^+$ T cells", *Annu. Rev. Immunol.* **12**, 635–673.

Siegrist, C.A. (2000) "Vaccination in the neonatal period and early infancy", *Int. Rev. Immunol.* **19**, 195–219.

Swain, S.L., Bradley, L.M., Croft, M., Tonkonogy, S., Atkins, G., Weinberg, A.D., Duncan, D.D., Hedrick, S.M., Dutton, R.W. and Huston, G. (1991) "Helper T-cell subsets: phenotype, function, and the role of lymphokines in regulating their development", *Immunol. Rev.* **123**, 115–144.

Tarlinton, D. (1998) "Germinal centers: form and function", *Curr. Opin. Immunol.* **10**, 245–251.

Yoshida, K. and Kikutani, H. (2002) "Genetic and immunological basis of autoimmune diabetes in the NOD mouse", *Rev. Immunogenet.* **2**, 140–146.

